# The N-type calcium channel rises from the ashes

**DOI:** 10.1172/JCI189308

**Published:** 2025-02-17

**Authors:** Maria A. Gandini, Gerald W. Zamponi

**Affiliations:** Department of Clinical Neurosciences, Hotchkiss Brain Institute and Alberta Children’s Hospital Research Institute, Cumming School of Medicine, University of Calgary, Calgary, Alberta, Canada.

## Abstract

Chronic pain is a debilitating condition that affects up to 1.5 billion people globally. Advancing pain management depends on a thorough understanding of the types of pain experienced by patients and the underlying mechanisms driving it. N-type calcium channels play a crucial role as therapeutic targets for managing chronic pain. In this issue of the *JCI*, Tang et al. introduce C2230, an N-type calcium channel blocker that inhibited Ca_V_2.2 channels during high frequency stimulation with little effect on other ion channels in the pain pathway across multiple models. C2230 offers a promising analgesic for use in therapeutic intervention.

## Drug development efforts for ion channel–blocking pain molecules

**Figure 1:**
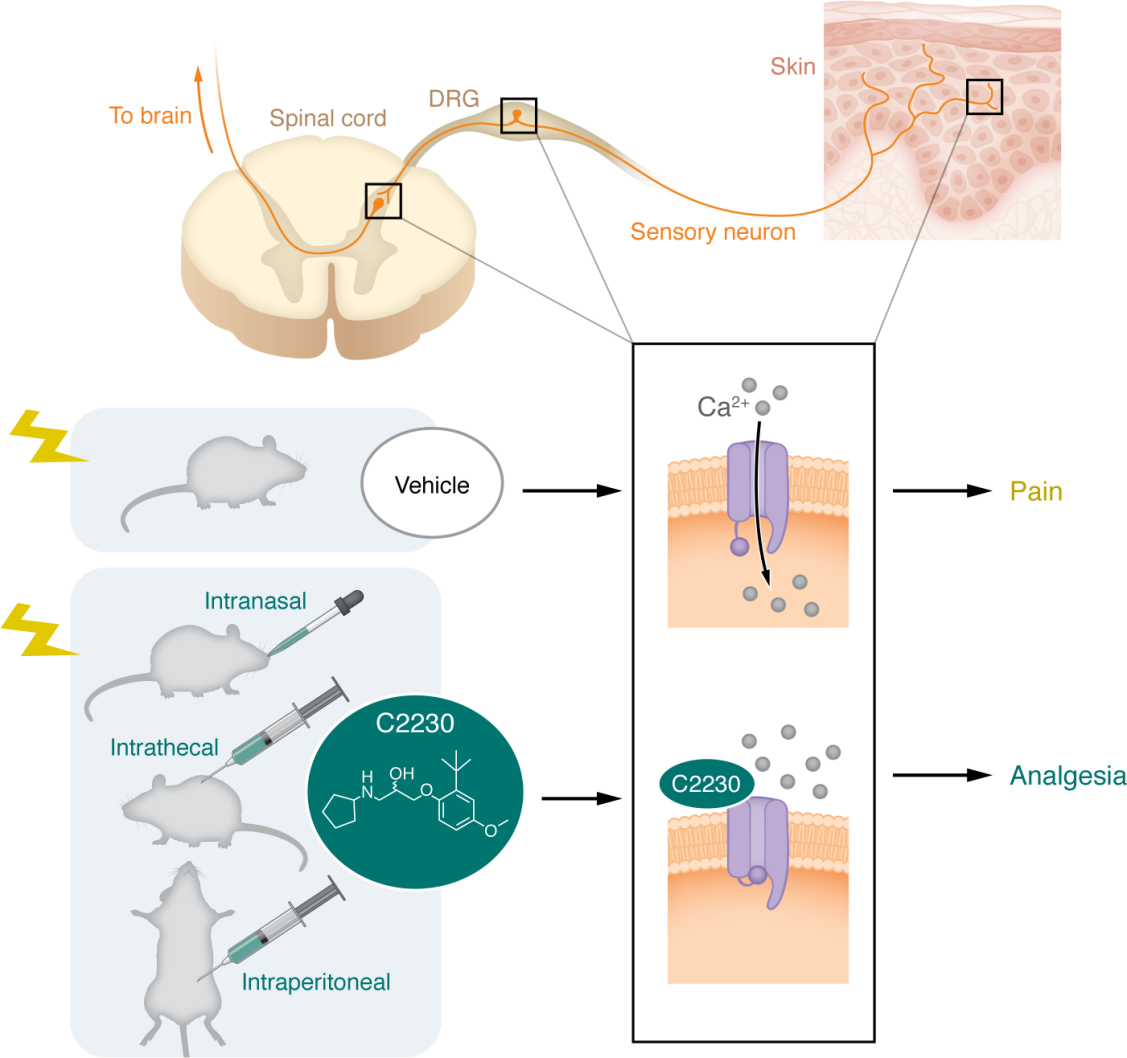
C2230 blocks N-type calcium channel and inhibits pain. N-type calcium channels are expressed in the primary afferent pathways that innervate the skin, originating from dorsal root ganglion (DRG) neurons and their synaptic nerve terminals in the spinal dorsal horn. C2230 given to rodents using any of three different routes prevented calcium entry via Ca_V_2.2 N-type calcium channels. The treatment led to analgesia of pain in three pain models.

N-type calcium channels are essential for neurotransmission at afferent sensory neuron synapses in the spinal dorsal horn. These channels are macromolecular complexes comprising the pore-forming Ca_V_2.2α1 subunit and auxiliary Ca_V_β and Ca_V_α2δ subunits, which regulate their trafficking and biophysical properties (1). In rodents, upon peripheral injury, there is an increase in the expression of Ca_V_2.2 and Ca_V_α2δ, and this change is thought to contribute to the heightened transmission of nociceptive inputs, and, consequently, pain. Gabapentinoids, such as gabapentin (Neurontin) and pregabalin (Lyrica), alleviate pain by interfering with the function of Ca_V_α2δ, thereby reducing the trafficking of N-type channels to synaptic terminals (2, 3). Furthermore, the highly selective N-type channel blocker ω-conotoxin MVIIA (a.k.a. Prialt as the synthetic version), a peptide isolated from the venom of the marine fish–hunting mollusk *Conus magus*, is approved for the treatment of severe refractory chronic pain (4). However, as this peptide does not cross the blood-brain barrier, it must be delivered intrathecally via an implanted mini pump, thus limiting its clinical utility (5). Consequently, efforts to develop small, orally bioavailable N-type calcium channel blockers began over two decades ago (6–10). Despite initial promise, inhibitors like Trox-1 (Merck), CNV2197944 (Convergence), and Z160 (Zalicus) advanced to clinical trials but were ultimately abandoned. Subsequently, drug development efforts for ion channel–blocking pain molecules shifted away from N-type channel inhibitors to blockers of T-type calcium channels and Nav1.7 and Nav1.8 sodium channels (11–13). An exciting study by Tang and colleagues published in this issue of the *JCI* (14) reports the identification and analgesic properties of a state-dependent Ca_V_2.2 channel blocker of N-type currents (C2230), potentially ushering in a second coming of N-type calcium channels as a drug target for pain (Figure 1).

## N-type calcium channels as a target

Tang and authors screened a compound library for inhibitors of Ca_V_2.2 calcium channels using patch clamp electrophysiology and identified a number of potential hit molecules, including compound C2230. The compound was then synthesized as a racemic mixture and subjected to a more detailed analysis using single cell patch clamp. C2230 potently blocked Ca_V_2.2 channels expressed exogenously in HEK cells, native N-type currents in rat and marmoset trigeminal neurons, as well as native N-type currents in human nociceptors. It had much less effect on other types of ion channels expressed in the pain pathway, including sodium and potassium channels, as well as on other calcium channel isoforms, overall indicating preferential inhibition of N-type channels. Block of N-type calcium channels occurred in a state-dependent manner, with inhibition favoring the inactivated channel conformation (Figure 1). This finding is important because inactivated channel block is predicted to result in use or frequency-dependent inhibition of the channel, as was clearly demonstrated by the authors. Indeed, binding to the inactivated conformation may be a desirable property for a channel blocker that targets a hyperexcitable neuronal state such as pain. Notably, in rat nociceptors, C2230 did not alter neuronal excitability per se and had no effect on the resting membrane potential, rheobase, or fast afterhyperpolarization, which is a term that describes a phase after an action potential. Instead, the compound was shown to reduce the strength of synaptic transmission as well as CGRP release from presynaptic terminals, consistent with an action on presynaptic calcium influx via N-type channels.

The therapeutic potential of C2230 was evaluated in three distinct pain models. In a spinal nerve ligation model, characterized by mechanical and cold hypersensitivity, systemic or intrathecal administration of C2230 reduced hypersensitivity without the development of tolerance following repeated dosing. Treated animals also showed dramatically reduced aversion to painful mechanical stimuli. Remarkably, in a model of chronic constriction injury of the infraorbital nerve, intranasal delivery of C2230 produced robust antinociceptive effects. These data suggest that compound C2230 may be effective against orofacial pain. Lastly, in an osteoarthritis-like pain model, C2230 effectively alleviated pain symptoms. Importantly, C2230 showed no effect on locomotor activity or evidence of toxicity.

The authors further extended their study to show that C2230 altered functional changes in the parabrachial nucleus that are known to occur during neuropathic pain conditions, suggesting that interfering with synaptic transmission at the level of the afferent pain pathway can prevent plasticity changes at higher CNS centers. Going hand-in-hand with these observations, Tang and colleagues showed that C2230 inhibited the aversive component of neuropathic pain in neuropathic rats. Finally, to conclude their tour de force, the authors performed a molecular docking analysis of compound C2230 to the Ca_V_2.2 channel and verified their in silico analysis with site-directed mutagenesis studies — these latter set of experiments may ultimately allow further refinement of C2230 to increase affinity and channel subtype specificity.

## Clinical implications

During pain states, neurons exhibit increased firing and heightened excitability, making N-type calcium channel state-dependent blockers a compelling approach for chronic pain management. However, two other use-dependent N-type blockers, CNV2197944 and Z160, advanced to phase II clinical trials but failed to outperform the placebo on any endpoint. Despite their potent use-dependent inhibition of N-type channels at nanomolar concentrations and favorable safety profiles in preclinical trials, the reason for their failure remains unclear. These challenges highlight the critical need to understand how C2230 can overcome similar problems and deliver on its therapeutic promise. The observation that an analgesic effect was observed with intranasal delivery of the compound in an orofacial pain model is quite striking and opens the possibility for treatment of trigeminal neuralgia, a painful condition that is often refractory to treatment. Whether intranasally delivered C2230 acts at synaptic terminals in the trigeminal nucleus or at peripheral sites needs to be further elucidated. In this context, it is interesting to note that a recent study has implicated a pronociceptive role of N-type calcium channels at peripheral nerve endings (15, 16) (Figure 1). It will also be interesting to determine whether C2230 may mediate analgesic effects by targeting N-type channels at nerve endings by intradermal or transdermal delivery.

Several other Ca_V_2.2 channel blockers with demonstrated efficacy in animal pain models have been reported in the literature. For example, RD2, an orally available molecule initially developed to target oligomeric amyloid beta protein (Aβ) aggregates (17), competes with ziconotide for binding to N-type calcium channels at nanomolar concentrations. RD2 reversibly and nearly completely inhibits Ca_V_2.2 channels, effectively alleviating neuropathic pain in an inflammatory neuritis rat model (18). However, high doses are required to reduce pain in acute thermal response models. Importantly, RD2 does not affect heart rate or blood pressure (19) and has demonstrated tolerability in healthy human volunteers (20). Altogether, such studies indicate that N-type calcium channels remain a viable and promising target in the pursuit of effective pain management.

The identification of C2230 as a state- and use-dependent N-type channel blocker represents a notable advancement in the development of inhibitors for chronic pain management. By preferentially targeting hyperactive neurons during pain states, C2230 may address the challenges that limited the success of other N-type channel blockers, such as off-target effects or inadequate efficacy. Its systemic and intranasal administration provides analgesic effects without tolerance or adverse behavioral effects. Given this preliminary safety profile, these features makes it a promising candidate for further development. A deeper understanding of its pharmacokinetics, long-term safety, and effectiveness in clinical trials will be critical to determining whether it has the potential to emerge as a groundbreaking solution for chronic pain management.

## Version 1 02/17/2025

Electronic publication
